# Impact of Alcohol Dehydrogenase 7 Polymorphism and Alcohol Consumption on Risk of Head and Neck Squamous Cell Carcinoma: A Korean Case-Control Study

**DOI:** 10.3390/jcm12144653

**Published:** 2023-07-13

**Authors:** Dong-Won Lee, Yong-Bae Ji, Chang-Myeon Song, Jeong-Kyu Kim, Seung-Hwan Lee, Kyung Tae

**Affiliations:** 1 Department of Otolaryngology-Head and Neck Surgery, School of Medicine, Daegu Catholic University, 33 Duryugongwon-ro 17-gil, Nam-gu, Daegu 42472, Republic of Korea; neck@cu.ac.kr (D.-W.L.); doctorjkkim@cu.ac.kr (J.-K.K.); 2 Department of Otolaryngology-Head and Neck Surgery, College of Medicine, Hanyang University, 222 Wangsimni-ro, Seongdong-gu, Seoul 04763, Republic of Korea; jyb20000@hanmail.net (Y.-B.J.); songabcd@hanmail.net (C.-M.S.); shleemd@hanyang.ac.kr (S.-H.L.)

**Keywords:** genetic susceptibility, molecular epidermiology, head and neck cancer, single nucleotide polymorphism, SNPs

## Abstract

Background: Head and neck squamous cell carcinoma (HNSCC) is closely associated with alcohol consumption and individual genetic susceptibility, such as single nucleotide polymorphism (SNP) of alcohol dehydrogenase (ADH). This study aimed to investigate the association of ADH7 SNPs with the risk of HNSCC. Methods: We analyzed ADH7 rs1573496C>G, rs3737482T>C, rs1154460G>A, and rs284787T>C SNPs in 250 patients with HNSCC and 322 controls in the Korean populations. Genotyping was conducted using the TaqMan assay. Linkage disequilibrium and haplotypes were analyzed. Results: The odds ratios (OR) and 95% confidence intervals (CI) of the CT and CC genotypes of ADH7 rs3737482T>C were 0.48 (0.29–0.78) and 0.69 (0.49–0.96), indicating a significantly decreased risk. In SNP of rs1154460G>A, the OR and 95% CI of the AA genotype was 1.63 (1.11–2.40), showing a significant increase in the risk. Furthermore, SNPs of ADH7 rs3737482T>C and ADH7 rs1154460G>A exhibit synergistic interactions with alcohol composition on the risk of HNSCC. None of the haplotypes were associated with the risk of HNSCC. Conclusions: ADH7 rs3737482T>C and rs1154460G>A SNPs are associated with the risk of development of HNSCC in Koreans. They could serve as molecular biological markers to screen high-risk groups for HNSCC.

## 1. Introduction

Alcohol consumption is closely associated with the development of head and neck squamous cell carcinoma (HNSCC) [1,2,3]. Many previous case-control studies and prospective research works reported that a certain amount of daily alcohol intake increases the risk of cancer in the oral cavity, pharynx, larynx, and esophagus [4,5]. The International Agency for Research on Cancer (IARC) concluded that alcohol was confirmed as a causative factor in the development of oral cavity, pharynx, larynx, esophagus, breast, liver, and colon cancer [4].

Ingested ethanol is oxidized to acetaldehyde mainly by alcohol dehydrogenase (ADH), and acetaldehyde is oxidized again to non-toxic acetate by aldehyde dehydrogenase (ALDH). Among these, acetaldehyde has been reported to play an important role in inducing tumors [6,7,8]. Also, the acetaldehyde concentration is found to be 10–20 times higher in saliva than in blood after drinking alcohol, which is considered to be associated with head and neck cancer [9].

Although alcohol consumption is the important risk factor for the development of HNSCC, host-related factors such as genetic polymorphism, which depends on the genetic differences of individuals, are also risk factors [10,11]. Altered genotypes of ADH, which may be responsible for the rapid metabolism of ethanol to acetaldehyde, exhibit a higher level of acetaldehyde in the body and, eventually, exhibit an increased risk of malignant tumors [12,13,14,15]. There are seven isoenzymes in five classes partitioned according to nucleotide sequence similarity: ADH1A, ADH1B, ADH1C in class I, ADH4 in class II, ADH5 in class III, ADH7 in class IV, and ADH6 in class V [16].

Most of the studies on ADH polymorphism of head and neck cancer have been about ADH1B and ADH1C polymorphism [12,13,14,15,17,18]. Studies on ADH7 SNP have been rare. Only a few studies exist on the association between ADH7 SNPs and the risk of head and neck cancer [19,20,21,22,23]. Even then, these studies show conflicting results between different ethnicities [19,20,21,22,23]. In addition, there has been no study on ADH7 SNPs of head and neck cancer in South Korea. Therefore, in this study, we investigated the SNPs of ADH7 and their relationship with the risk of HNSCC in Koreans.

## 2. Materials and Methods

### 2.1. Subjects

Two hundred and eighty patients with HNSCC who were treated in the Department of Otolaryngology and Head and Neck Surgery at Hanyang University Hospital from January 2000 to December 2004 were enrolled in this study as the cases. The control group included 330 patients who were hospitalized during the same period for chronic sinusitis, chronic otitis media, and chronic tonsillitis. Those with a history of malignant tumors at other sites or specific genetic disorders were excluded. We calculated the sample size using a two-sided test with the predicted odds ratio of 0.78, a significance level of 0.05, and a power of 80%. All the participants of this study were of Korean nationality and were living in South Korea at the time the study was carried out.

The average age of the case group was 62.2 (28–86 years) and 43.8 (21–76 years) in the control group, showing a higher average age in the case group than in the control group (*p* < 0.001). There were 219 males and 31 females in the case group and 305 males and 17 females in the control group. Significantly more heavy drinkers and smokers were found in the case group compared to the control group (*p* < 0.001) (Table 1). By primary sites of HNSCC, there were 120 patients with laryngeal cancer, 64 patients with oral cavity cancer, 31 patients with oropharyngeal cancer and, 35 patients with hypopharyngeal cancer.

The participants were divided according to their alcohol consumption history: non-drinkers and drinkers. Also, the drinker group was categorized into social drinkers who consumed alcohol at a rate of less than 120 g per week and heavy drinkers who consumed more than 120 g per week. In order to calculate weekly ethanol consumption, we used a standard conversion table for alcoholic beverages that considers 360 mL of soju to be equivalent to 49 g of ethanol, 180 mL of sake to 22 g, 500 mL of beer to 18 g, 30 mL of whiskey to 10 g, and 120 mL of wine to 11 g. The participants were also classified into non-smokers, light smokers with a smoking history of less than 20 pack-years (number of packs per day multiplied by number of years of smoking), and heavy smokers with a smoking history of 20 pack-years or more.

### 2.2. Methods

#### 2.2.1. DNA Extraction

A 10 mL sample of peripheral blood was collected in a tube containing EDTA, stored at −70 °C. From this sample, a 1 mL portion was used for the experiment. DNA from the peripheral blood was extracted using WizardTM Genomic DNA purification kit (Promega, Madison, WI, USA).

#### 2.2.2. Genetic Analysis

ADH7 rs1573496C>G, rs3737482T>C, rs1154460G>A, and rs284787 T>C were analyzed using the TaqMan assay method. The forward and reverse primers used in the experiments are shown in Table 2. Polymerase chain reaction (PCR) was performed using a 5 μL sample of the mixture made of TaqMan universal PCR master mix (Applied Biosystems, Foster City, CA, USA), UNG, 900 μM of each primer, 200 nM of each probe, and 20 ng of genomic DNA. The experiment was carried out at 50 °C for 2 min and at 95 °C for 10 min for reaction, followed by denaturing at 95 °C for 15 s, and annealing and extension at 60 °C for 1 min, which results in a 40-fold amplification. The TaqMan assay plate was moved to the Prism 9700HT instrument (Applied Biosystems), and the fluorescent intensity of each well was measured for the genetic analysis with SDS software ver. 2.3. (Applied Biosystems, Foster City, CA, USA).

#### 2.2.3. Haplotype Analysis

Linkage disequilibrium in gene analysis is a statistically inconsistent condition in which the frequencies present at two different genetic loci with a theoretical value that exists under the assumption that the two genetic loci are independent and not associated with each other in the group; meaning that they act as if they are related, regardless of whether or not they are the same gene.

Haplotype refers to a combination of series of SNPs that exists close to each other on one gene. They appear as a bundle, not independently but statistically associated with each other on one gene. It is easy to obtain information about the association of nucleotide polymorphism combinations; therefore, detailed information could be found compared to studying individual SNPs.

Linkage disequilibrium was analyzed using Haploview v4.2 software. Lewontin’s D′ (|D′|), and the LD coefficient r2 between all pairs of biallelic loci were examined. Haplotypes were estimated using PHASE v2.0 software from the University of Washington. In ADH7 SNPs, three types of haplotype, showing more than 5% of frequency observed in rs3737482T>C and rs1154460G>A, were analyzed.

#### 2.2.4. Statistics

For the statistical analyses of differences in demographic variables and ADH7 SNP between the HNSCC patients and the control group, the Student’s t-test was used for continuous variables, while the chi-square test and Fisher’s exact test were used for categorical variables. Odds ratio (OR) and 95% confidence interval (CI) for genetic polymorphisms were calculated using logistic regression analysis after adjusting for sex, age (continuous variable), smoking status, and alcohol consumption. Univariate analyses were performed using the referent model (heterozygotes vs. major homozygotes and minor homozygotes vs. major homozygotes) and three alternative models: (1) the co-dominant model (minor allele homozygotes vs. heterozygotes vs. major allele homozygotes); (2) the dominant model for minor alleles (minor allele homozygotes plus heterozygotes vs. major allele homozygotes); (3) the recessive model for minor alleles (minor allele homozygotes vs. heterozygotes plus major allele homozygotes). SPSS (version 18.0, SPSS Inc., Chicago, IL, USA) was used for statistical analysis. A *p*-value less than 0.05 was considered statistically significant.

## 3. Results

Of the 280 cases and 330 controls enrolled in the case and control groups, ADH7 genotyping was performed successfully in 250 of 280 participants in the case group and in 322 of 330 participants in the control group. We conducted a case-control study involving those participants with successful genotyping.

### 3.1. ADH7 Allele Frequency

The allele frequencies of ADH7 rs1573496C>G, rs3737482T>C, rs1154460G>A, and rs284787T>C were examined (Table 3). All genetic polymorphisms fit the Hardy–Weinberg equilibrium.

### 3.2. ADH7 Polymorphism and Risk of Head and Neck Squamous Cell Carcinoma

In ADH7 rs1573496C>G, the frequencies were 100% only in the CC genotype in the case group, and the frequencies of CC, CG, and GG genotypes were 99.7%, 0.3%, and 0%, respectively, in the control group. The odds ratio was undetermined (Table 4).

In ADH7 rs3737482T>C, the frequencies of the three genotypes TT, CT, and CC were 37.2%, 44.4%, 18.4%, respectively, in the case group and 25.2%, 52.5%, 22.4% in the control group, respectively (Table 4). Based on the TT genotype, the OR of the CT genotype was 0.48 (95% CI: 0.29–0.78), and the OR of the CC genotype was 0.69 (95% CI: 0.49–0.96), showing significantly low odds ratios in both genotypes.

In ADH7 rs1154460G>A, the frequencies of the three genotypes GG, AG, and AA were 37.6%, 48.0%, 14.4%, respectively, in the case group and 42.2%, 48.8%, 9.0%, respectively, in the control group (Table 4). Based on the GG genotype, the OR of the AG genotype was 1.47 (95% CI: 1.06–2.04) and 2.03 (95% CI: 1.05–3.92) in the co-dominant and recessive models, respectively, while the OR of the AA genotype was 1.63 (95% CI: 1.11–2.40) in the referent model, showing a significantly higher odds ratio in the AA genotype.

In ADH7 rs284787T>C, the frequencies of the three genotypes TT, CT, and CC were 40.4%, 47.2%, 12.4%, respectively, in the case group and 45.7%, 42.2%, 12.1%, respectively, in the control group (Table 4). Based on the TT genotype, the OR of the CT genotype was 1.43 (95% CI: 0.90–2.28), and the CC genotype was 1.24 (95% CI: 0.87–1.74). Neither genotype showed statistically significant differences.

### 3.3. Linkage Disequilibrium and Haplotype of ADH7

**Figure 1:**
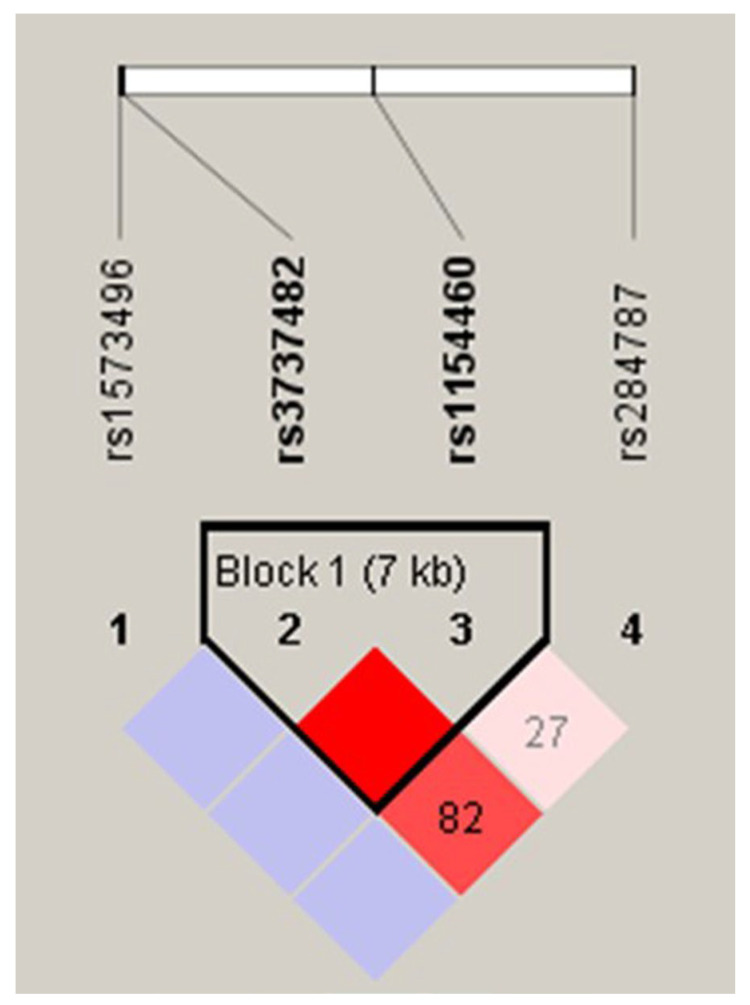
Gene structure and linkage disequilibrium of genotyped single nucleotide polymorphisms (SNPs) of AHD7. Linkage disequilibrium is displayed using Haploview. D’values are given in the rhombus cell intersecting for each pair of SNPs. A blank cell indicates D’ = 1.0. The darker the cell, the greater is the linkage disequilibrium between the SNPs. Haplotype blocks are outlined.

In the analysis of ADH7 linkage disequilibrium, high linkage disequilibrium occurred only between rs3737482T>C and rs1154460G>A (Figure 1). Three types of haplotype were found showing more than 5% of the frequency observed in ADH7 rs3737482T>C and rs1154460G>A. Of the three haplotypes, ADH7-ht1 and ADH7-ht2 were excluded from the analysis as they were almost identical, with rs3737482T>C and rs1154460G>A, respectively. The ADH7-ht3 haplotype (T allele in rs3737482 and G allele in rs1154460) was analyzed.

In the genetic analysis of ADH7 ht3, the ht3 −/− group was 62.0% in the case group and 67.1% in the control group. The ht3 +/− group was 34.0% in the case group and 29.8% in the control group. The ht3 +/+ group was 4.0% in the case group and 3.1% in the control group. Based on the ht3 −/− group, the OR of the ht3 +/− group was 1.22 (95% CI: 0.77–1.93) and 0.99 (95% CI: 0.55–1.78) in the ht3 +/+ group, and no statistically significant difference was found (Table S1).

### 3.4. ADH7 rs3737482T>C and rs1154460G>A and Risk of Head and Neck Squamous Cell Carcinoma According to Alcohol Consumption

The relative risk of HNSCC was analyzed in ADH7 rs3737482T>C and rs1154460G>A, according to alcohol drinking history. In ADH7 rs3737482T>C, based on the TT genotype, the OR was 0.43 (95% CI: 0.42–0.77) in the CC genotype and 0.42 (95% CI: 0.19–0.94) in the CT genotype in the drinker group, indicating a significant decrease. In ADH7 rs1154460G>A, based on the GG genotype, the OR of the AA genotype were 3.55 (95% CI: 1.51–8.33) in the drinker group, indicating a significant increase (Table 5). The analysis was also performed by dividing the drinker group into social drinkers and heavy drinkers. In ADH7 rs3737482T>C, based on the TT genotype, the OR was 0.16 (95% CI: 0.04–0.69) in the CC genotype in the social drinker group and 0.40 (95% CI: 0.17–0.92) in the CT genotype in the heavy-drinker group (Table S2). In ADH7 rs1154460G>A, based on the GG genotype, the OR of the AG genotype and the AA genotype were 2.60 (95% CI: 1.02–6.62) and 5.85 (95% CI: 1.58–21.62) in the social drinker group (Table S3).

## 4. Discussion

In terms of ADH7 SNPs in head and neck cancer, ADH7 rs1573496 SNP has been most commonly investigated. Several studies analyzed the relationship between the ADH7 rs1573496 SNP and the risk of HNSCC [19,20,21,22,23,24,25]. However, conflicting results are found regarding the relationship between the ADH7 rs1573496 SNP and the risk of HNSCC among previous studies. Hashibe et al. analyzed 3800 patients with malignant tumors of the upper areodigestive tract, including esophageal cancer, and 5200 of the control group from three multi-center data sources in Europe and Latin America. They reported that the CC genotype of ADH7 rs1573496C>G exhibits an OR of 0.68 (95% CI: 0.60–0.78), showing a protective effect against cancer [24]. Wei et al. performed a case-control study on 1110 patients with HNSCC and 1,129 controls in non-Hispanic white Americans. They reported that the OR of the GG genotype in ADH7 rs1573496C>G was 0.32 (95% CI: 0.13–0.82), and the OR of CG+GG genotype was 0.74 (95% CI: 0.59–0.94), indicating a decreased risk of head and neck cancer [20].

However, some studies failed to detect any significant correlation between ADH7 rs1573496 SNP and the risk of HNSCC. Hakenewerth et al. reported that ADH7 rs1573496 SNP did not show a clear relationship with head and neck cancer risk in European-American and African-American patients with HNSCC [21]. In addition, another study conducted in Japan using 585 patients with head and neck cancer and 1170 controls reported that ADH7 rs1573496 SNP was comprised of all homozygous C alleles, and further analysis was not possible [22]. Also, the other study conducted in India to evaluate the relationship between oral cancer and alcohol metabolism revealed the rarity of ADH7 rs1573496 in the Indian population [23]. In the current study, all HNSCC patients showed CC homozygous in ADH7 rs1573496C>G, and only one patient in the control group exhibited the CG genotype. Therefore, performing further analysis was not possible. These results suggest that the frequencies of the alleles and SNP genotypes of ADH7 rs1573496 vary by different ethnic groups.

Concerning ADH7 rs1154460G>A, previous studies showed differences based on ethnicity. Hakenewerth et al. reported that ADH7 rs1154460G>A SNP did not show a clear relationship with head and neck cancer risk in European-American and African-American patients with HNSCC [21]. This is in contrast to the study that investigated this allele and found it to be associated with an increased OR in Japanese populations [22]. In the current study, ADH7 rs1154460G>A SNP was associated with an increased risk of head and neck cancer.

Regarding ADH7 rs3737482T>C and rs284787T>C SNPs in head and neck cancer, the results of the current study were similar to those of a previous study conducted by Oze et al., confirming the association with the risk of head and neck cancer [22]. Oze et al. reported that ADH7 rs3737482T>C SNP results in an independent and significant effect on decreasing the risk of head and neck cancer [22]. In addition, in the current study, ADH7 rs3737482T>C SNP was associated with decreased risk of head and neck cancer. Regarding ADH7 rs284787T>C SNP, Oze et al. showed no statistically significant result [22]. Also, in this study, it was not associated with the risk of HNSCC.

Determining the effects of individual polymorphism on cancer is not easy because all ADH genes are in one cluster, and linkage disequilibrium exists. Recent multi-center research conducted by Hashibe et al. using HapMap analysis suggested that ADH1A, ADH1B, ADH1C, ADH4, ADH5, and ADH6 exhibit a relatively high linkage disequilibrium, but ADH7 exhibits relatively smaller linkage disequilibrium compared to the other six types [24].

In this study, the haplotype results showed no independent association with HNSCC risk. This might be due to the effects of the strong linkage disequilibrium between ADH7 rs3737482 and rs1154460 SNPs.

A possible interaction between environmental factors and the genetic susceptibility for cancer has been suggested. Hakenewerth et al. reported a synergistic interaction between alcohol consumption and genetic polymorphism in head and neck cancer development [21]. In the current study, we analyzed HNSCC risk changes in ADH7 rs3737482T>C and rs1154460G>A SNPs, according to alcohol consumption, to evaluate the interaction between genetic polymorphism and environmental factors. No statistically significant interaction was found in both SNPs in the non-drinker group. However, in the drinker group, the OR of rs3737482T>C decreased significantly, and the OR of rs1154460G>A increased significantly. These results might support a synergistic interaction between the environmental factor of alcohol consumption and the genetic factor of ADH7 SNP. However, the OR did not increase in proportion to the alcohol consumption in the social or heavy-drinker groups. A small sample size might be related to these heterogeneous results. Also, one study that investigated the association between five ADH1B-ADH1C-ADH7 cluster SNPs and the risk of developing esophageal squamous cell carcinoma in the Chinese population did not show any significant gene-drinking interaction [26]. Therefore, further studies with large sample sizes in different ethnic groups may be necessary to clarify the effect of the interaction between ADH7 SNPs and alcohol consumption on the risk of HNSCC. Investigating enzyme function and activity according to SNPs and alcohol consumption is also necessary.

The current study exhibits some limitations. First, it was a hospital-based case-control study; the control group was made up of non-cancer patients who visited the hospital. Therefore, an evitable bias and a question exists; whether the gene frequency of the control group could represent the general population, although the genotype frequencies did not deviate from the Hardy–Weinberg equilibrium in the control group. Second, the sample size of the study might be relatively too small to reach a firm conclusion. Third, a significant difference was found in the mean age between the case and control groups. This might influence the study results, although the OR was analyzed with adjustment for age. Further studies with large sample sizes and age-matched samples would have more strength and allow for better risk estimation. Fourth, information regarding other risk factors such as HPV infection for developing HNSCC is lacking. Fifth, we acknowledge that our inability to link particular SNPs and combinations of SNPs to ADH7 alleles and/or maternal or paternal inheritance does limit our findings. The missing knowledge about how these SNPs are involved in regulating alcohol dehydrogenase activity and the development of HNSCC, particularly in the context of the genetic background of Korean patients, represents an important area for further research.

Despite these limitations, it is of note that this study is the first study to have analyzed the four SNPs of ADH7 and assess their relationship with the risk of developing HNSCC in Koreans.

## 5. Conclusions

Based on the results of the current study, SNPs of ADH7 rs3737482T>C and ADH7 rs1154460G>A are associated with the risk of the development of head and neck squamous cell carcinoma in Koreans and they could be potentially useful molecular biological markers for identifying individuals at high risk of developing HNSCC. Furthermore, SNPs of ADH7 rs3737482T>C and ADH7 rs1154460G>A exhibit synergistic interactions with alcohol composition on the risk of HNSCC. 

## Figures and Tables

**Table 1:** Demographic characteristics of the study population (*n* = 572).

Variables	Case		Control		*p*-Value
	N	%	N	%	
Age (year)					<0.001
≤45	23	(9.2)	197	(61.2)	
45–60	73	(29.2)	88	(27.3)	
≥60	154	(61.6)	37	(11.5)	
Sex					0.002
Male	219	(87.6)	305	(94.7)	
Female	31	(12.4)	17	(5.3)	
Smoking					<0.001
None	43	(17.2)	1	(0.3)	
Light *	44	(17.6)	82	(25.5)	
Heavy ^†^	163	(65.2)	239	(74.2)	
Alcohol status					<0.001
None	93	(37.2)	89	(27.6)	
Social ^‡^	53	(21.2)	149	(46.3)	
Heavy ^§^	104	(41.6)	84	(26.1)	
	250		322		

* less than 20 pack-years; ^†^ 20 pack-years or more; ^‡^ Drink less than 120 g of alcohol per week; ^§^ Drink 120 g of alcohol or more per week.

**Table 2:** Primer sequence for ADH7 single nucleotide polymorphism genotyping.

Gene	Locus	Primer Name	Primer Sequence
*ADH7*	*rs1573496_CG*	rs1573496_CG_F	TCGCTCCTAATGCAAAGGTT
		rs1573496_CG_R	CAGATTTTGGCCACAGGAAT
		rs1573496_CG_AM21	CCTGGTTTCACTGTAGTCACT
	*rs3737482 _TC*	rs3737482 _TC_F	TCGCTCCTAATGCAAAGGTT
		rs3737482 _TC_R	CAGATTTTGGCCACAGGAAT
		rs3737482 _TC_AM27	TCTAAGGTTTATAAACACCGTAAGGTT
	*rs1154460_GA*	rs1154460_GA_F	TTTGGTCCAGTGAACCTGCT
		rs1154460_GA_R	ATTGGGCATCTTGAAACCAT
		rs1154460_GA_AM38	ATAATAAAGTTATAGACAATTAAATTTACCTTGTGCAT
	*rs284787_TC*	rs284787_TC_F	ACAAGGCTATTTGCCAGCAT
		rs284788_TC_R	AAAAACACTTTTTATTAAATGGAGTCA
		rs284789_TC_AM32	TGTTCAATTTGATACAGTAGAATTGCAAGTCC

**Table 3:** Single nucleotide polymorphism and allele frequencies of ADH7 in Korean head and neck squamous cell carcinoma patients and controls.

Gene	Locus	Amino Acid Change	Genotype				Frequency	HWE *
*ADH7*	rs1573496C>G	Gly92Ala	C	CG	G	N	0.001	0.983
			571	1	0	572		
	rs3737482T>C		T	CT	C	N	0.451	0.783
			174	280	118	572		
	rs1154460G>A		G	AG	A	N	0.356	0.177
			230	277	65	572		
	rs284787T>C		T	CT	C	N	0.344	0.690
			248	254	70	572		

* *p*-value for deviation from Hardy–Weinberg Equilibrium.

**Table 4:** Logistic analysis of ADH7 single nucleotide polymorphisms in Korean head and neck squamous cell carcinoma patients and controls.

Loci	GenoType	Distribution		Referent Analysis		Codominant Analysis		Dominant Analysis		Recessive Analysis	
		Case (%)	Control (%)	OR * (95% CI ^†^)	*p*	OR (95% CI)	*p*	OR (95% CI)	*p*	OR (95% CI)	*p*
rs1573496C>G	CC	250 (100.0)	321 (99.7)	1							
	CG	0 (0.0)	1 (0.3)								
	GG	0 (0.0)	0 (0.0)								
rs3737482T>C	TT	93 (37.2)	81 (25.2)	1							
	CT	111 (44.4)	169 (52.5)	0.48 (0.29–0.78)	0.003	0.66 (0.49–0.90)	0.008	0.47 (0.30–0.75)	0.002	0.76 (0.44–1.29)	0.31
	CC	46 (18.4)	72 (22.4)	0.69 (0.49–0.96)	0.03						
rs1154460G>A	GG	94 (37.6)	136 (42.2)	1							
	AG	120 (48.0)	157 (48.8)	1.31 (0.83–2.06)	0.25	1.47 (1.06–2.04)	0.02	1.49 (0.96–2.32)	0.08	2.03 (1.05–3.92)	0.04
	AA	36 (14.4)	29 (9.0)	1.63 (1.11–2.40)	0.01						
rs284787T>C	TT	101 (40.4)	147 (45.7)	1							
	CT	118 (47.2)	136 (42.2)	1.43 (0.90–2.28)	0.13	1.32 (0.97–1.80)	0.08	1.47 (0.95–2.28)	0.08	1.38 (0.74–2.58)	0.32
	CC	31 (12.4)	39 (12.1)	1.24 (0.87–1.74)	0.23						

* Adjusted odds ratio; ^†^ 95% Confidence interval.

**Table 5:** Logistic analysis of ADH7 rs3737482T>C and rs1154460G>A polymorphism in Korean head and neck squamous cell carcinoma patients and controls according to alcohol consumption.

Gene	Alcohol	Genotype	Cancer	Normal	OR * (95% CI ^†^)	*p*
*ADH7* rs3737482T>C	Non-drinker (*n* = 182)	TT	26 (28.0%)	21 (23.6%)	1	
		CT	44 (47.3%)	42 (47.2%)	0.59 (0.21–1.68)	0.322
		CC	23 (24.7%)	26 (29.2%)	0.75 (0.23–2.42)	0.628
	Drinker (*n* = 390)	TT	67 (42.7%)	60 (25.8%)	1	
		CT	67 (42.7%)	127 (54.5%)	0.43 (0.42–0.77)	0.004
		CC	23 (14.6%)	46 (19.7%)	0.42 (0.19–0.94)	0.034
*ADH7* rs1154460G>A	Non-drinker (*n* = 182)	GG	43 (46.2)	41 (46.1)	1	
		AG	39 (41.9)	39 (43.8)	0.80 (0.32–2.01)	0.631
		AA	11 (11.8)	9 (10.1)	1.25 (0.30–5.27)	0.760
	Drinker (*n* = 390)	GG	51 (32.5%)	95 (40.8%)	1	
		AG	81 (51.6%)	118 (50.6%)	1.55 (0.87–2.75)	0.136
		AA	25 (15.9%)	20 (8.6%)	3.55 (1.51–8.33)	0.004

* Adjusted odds ratio; ^†^ 95% Confidence interval.

## Data Availability

The data presented in this study are available on request from the corresponding author.

## Supplementary Materials

The following supporting information can be downloaded at: https://www.mdpi.com/article/10.3390/jcm12144653/s1, Table S1: Analysis of haplotypes of ADH7 in Korean head and neck squamous cell carcinoma patients and controls. Table S2: Logistic analysis of ADH7 rs3737482T>C polymorphism in Korean head and neck squamous cell carcinoma patients and controls according to alcohol consumption. Table S3: Logistic analysis of ADH7 rs1154460G>A polymorphism in Korean head and neck squamous cell carcinoma patients and controls according to alcohol consumption.
