# Supplementary material for: Impact of Alcohol Dehydrogenase 7 Polymorphism and Alcohol Consumption on Risk of Head and Neck Squamous Cell Carcinoma: A Korean Case-Control Study
Source: J Clin Med. 2023 Jul 13;12(14):4653. doi: 10.3390/jcm12144653 (PMC10380624; doi:10.3390/jcm12144653)
Supplement: Supplementary file 1 [file jcm-12-04653-s001.zip › Table S1.pdf]

Table S1. Analysis of haplotypes of *ADH7* in Korean head and neck squamous cell carcinoma patients and controls.

Gene	Loci	Genotype	Distribution		Referent analysis		Codominant analysis		Dominant analysis		Recessive analysis	
			Case (%)	Control (%)	OR* (95% CI†)	*p*	OR (95% CI)	*p*	OR (95% CI)	*p*	OR (95% CI)	*P*
ADH7	ht3	-/-	155 (62.0)	216 (67.1)	1							
		ht3/-	85 (34.0)	96 (29.8)	1.22 (0.77-1.93)	0.40	1.12 (0.77-1.65)	0.55	1.19 (0.76-1.86)	0.45	0.91 (0.29-2.87)	0.88
		ht3/ht3	10 (4.0)	10 (3.1)	0.99 (0.55-1.78)	0.97						

* Adjusted odds ratio; †95% Confidence interval
