# Supplementary material for: Impact of Alcohol Dehydrogenase 7 Polymorphism and Alcohol Consumption on Risk of Head and Neck Squamous Cell Carcinoma: A Korean Case-Control Study
Source: J Clin Med. 2023 Jul 13;12(14):4653. doi: 10.3390/jcm12144653 (PMC10380624; doi:10.3390/jcm12144653)
Supplement: Supplementary file 1 [file jcm-12-04653-s001.zip › Table S2.pdf]

Table S2. Logistic analysis of *ADH7* rs3737482T>C polymorphism in Korean head and neck squamous cell carcinoma patients and controls according to alcohol consumption.

Alcohol	Genotype	Cancer	Normal	OR* (95% CI†)	*P*
Non-drinker (n=182)	TT	26 (28.0 %)	21 (23.6 %)	1	
	CT	44 (47.3 %)	42 (47.2 %)	0.59 (0.21-1.68)	0.322
	CC	23 (24.7 %)	26 (29.2 %)	0.75 (0.23-2.42)	0.628
Social drinker (n=202)	TT	23 (43.4 %)	38 (25.5 %)	1	
	CT	26 (49.1 %)	82 (55.0 %)	0.53 (0.22-1.27)	0.151
	CC	4 (7.5 %)	29 (19.5 %)	0.16 (0.04-0.69)	0.014
Heavy drinker (n=188)	TT	44 (42.3 %)	22 (26.2 %)	1	
	CT	41 (39.4 %)	45 (53.6 %)	0.40 (0.17-0.92)	0.032
	CC	19 (18.3 %)	17 (20.2 %)	0.72 (0.25-2.10)	0.549

* adjusted Odds ratio; †95% Confidence interval
