# Supplementary material for: Impact of Alcohol Dehydrogenase 7 Polymorphism and Alcohol Consumption on Risk of Head and Neck Squamous Cell Carcinoma: A Korean Case-Control Study
Source: J Clin Med. 2023 Jul 13;12(14):4653. doi: 10.3390/jcm12144653 (PMC10380624; doi:10.3390/jcm12144653)
Supplement: Supplementary file 1 [file jcm-12-04653-s001.zip › Table S3.pdf]

Table S3. Logistic analysis of *ADH7* rs1154460G>A polymorphism in Korean head and neck squamous cell carcinoma patients and controls according to alcohol consumption.

Alcohol	Genotype	Case (%)	Control (%)	OR* (95% CI†)	*P*
Non-drinker (n=182)	GG	43 (46.2)	41 (46.1)	1	
	AG	39 (41.9)	39 (43.8)	0.80 (0.32-2.01)	0.631
	AA	11 (11.8)	9 (10.1)	1.25 (0.30-5.27)	0.760
Social drinker (n=202)	GG	13 (24.5)	60 (40.3)	1	
	AG	30 (56.6)	75 (50.3)	2.60 (1.02-6.62)	0.046
	AA	10 (18.9)	14 (9.4)	5.85 (1.58-21.62)	0.008
Heavy drinker (n=188)	GG	38 (36.5)	35 (41.7)	1	
	AG	51 (49.0)	43 (51.2)	1.05 (0.48-2.32)	0.903
	AA	15 (14.4)	6 (7.1)	2.79 (0.77-10.13)	0.119

* adjusted Odds ratio; †95% Confidence interval
